# Function Prediction and Analysis of *Mycobacterium tuberculosis* Hypothetical Proteins

**DOI:** 10.3390/ijms13067283

**Published:** 2012-06-13

**Authors:** Gaston K. Mazandu, Nicola J. Mulder

**Affiliations:** Computational Biology Group, Department of Clinical Laboratory Sciences, Institute of Infectious Disease and Molecular Medicine, University of Cape Town, Cape Town 7925, South Africa; E-Mail: gmazandu@gmail.com

**Keywords:** hypothetical protein, tuberculosis, genome analysis, function prediction, interaction network

## Abstract

High-throughput biology technologies have yielded complete genome sequences and functional genomics data for several organisms, including crucial microbial pathogens of humans, animals and plants. However, up to 50% of genes within a genome are often labeled “unknown”, “uncharacterized” or “hypothetical”, limiting our understanding of virulence and pathogenicity of these organisms. Even though biological functions of proteins encoded by these genes are not known, many of them have been predicted to be involved in key processes in these organisms. In particular, for *Mycobacterium tuberculosis*, some of these “hypothetical” proteins, for example those belonging to the Pro-Glu or Pro-Pro-Glu (PE/PPE) family, have been suspected to play a crucial role in the intracellular lifestyle of this pathogen, and may contribute to its survival in different environments. We have generated a functional interaction network for *Mycobacterium tuberculosis* proteins and used this to predict functions for many of its hypothetical proteins. Here we performed functional enrichment analysis of these proteins based on their predicted biological functions to identify annotations that are statistically relevant, and analysed and compared network properties of hypothetical proteins to the known proteins. From the statistically significant annotations and network information, we have tried to derive biologically meaningful annotations related to infection and disease. This quantitative analysis provides an overview of the functional contributions of *Mycobacterium tuberculosis* “hypothetical” proteins to many basic cellular functions, including its adaptability in the host system and its ability to evade the host immune response.

## 1. Introduction

Despite ever-increasing amounts of biological data, including primary data, such as genomic sequences, and functional genomic data from high-throughput experiments, there is a deficiency in functional annotation for many newly sequenced proteins. For instance, in most bacterial genomes, as many as 40% of identified proteins are labeled “uncharacterized” or “unknown” or “hypothetical” proteins [1]. Specifically, about half of the *Mycobacterium tuberculosis* genome is made up of proteins of unknown functions. This limits the ability to exploit these data, confirming the paradigm of “a world which is data rich yet information poor” where the deluge of data is caught by the need for efficient computational methods to extract information from these data. Thus, one of the major tasks in the post-genomic era is genome annotation, assigning functions to gene products based mostly on amino acid sequence, in order to capitalize on the knowledge gained through these sequencing efforts [2]. To this end, controlled vocabulary and well-defined protein function relationship schemes arose to represent annotations of known genes and proteins, and to predict functional annotations of those which are identified but so far uncharacterized. The terms used for describing a function should have definitions and be placed within a structure of relationships in an ontology [3].

An ontology is an explicit specification of concepts that includes a set of objects, their properties and their values, along with describable relationships between them. This is reflected in a representational vocabulary for a specific domain, containing definitions of classes, relations, functions and other objects [4–8]. The Gene Ontology (GO) [9,10] is one of the greatest contributions to the area of functional annotations. It is the most widely adopted ontology by the life science community and currently serves as the dominant and most popular functional classification scheme for functional representation and annotation of genes and their products. The GO annotation (GOA-UniProtKB) project arose in order to provide high-quality annotations to gene products, and is applied in the UniProt knowledgebase (UniProtKB) [11–14]. It also provides a central dataset for annotation in other major multi-species databases, such as Ensembl and NCBI [15]. Most of the annotations (approximately 98%) in the GOA dataset have been inferred electronically, with the IEA (Inferred from Electronic Annotation) GO evidence code, but are of high quality [16,17]. They can also be inferred from sequence homology searches, where functions of proteins of known function are assigned to a query protein using sequence similarity search tools, such as the Basic Local Alignment Search Tool (BLAST) [18,19]. This approach is referred to as homology-based annotation transfer and offers an easy and effective scheme for suggesting possible functions for proteins under consideration, but its applicability is limited and has thus left several proteins uncharacterized.

Proteins perform an astonishing range of biological functions in an organism. These include roles as structural proteins, enzymes and for the transportation of materials within and between cells [20]. Each protein is a gene product that interacts with the cellular environment in some way to promote the cell’s growth and function [21]. This suggests that these large numbers of uncharacterized proteins in organisms may play a crucial role in their survival and viability or may contribute to their fitness in different environments, particularly since uncharacterized proteins are usually those that have no hits in other genomes and are thus unique to certain organisms. Specifically for *Mycobacterium tuberculosis* (MTB), some of these “hypothetical” proteins, for example those belonging to the Pro-Glu or Pro-Pro-Glu (PE/PPE) family, are generally uncharacterized and their subcellular location is unknown. These proteins have sequences with characteristic motifs Pro-Glu at positions 8–9 and Pro-Pro-Glu at 8–10 [22], where Pro and Glu stand for Proline (P) and Glutamic (E) amino acids. They have been suspected to form a source of antigenic variation among different strains of MTB [23] and might interfere with immune responses by inhibiting antigen processing [24]. Some annotation predictions of these proteins indicate that they are expressed based on the changing micro-environments encountered by the pathogen and play an important role in survival and multiplication of MTB in their chosen environment, and even in mediating mycobacterium-host cell interactions [25–27]. This is also a highly expanded family of proteins, thus knowing the functions of these proteins is important for enhancing our understanding of this organism.

The biological analysis of organisms has evolved from the single gene approach to a whole genome focus, providing the opportunity to look at genes within their context in a cell [28]. The quantity of biological data has grown exponentially as a result of worldwide DNA sequencing efforts and high-throughput biology technologies. Integration of these vast amounts of data and identifying functional connections between characterized and hypothetical proteins have the potential to facilitate functional annotation of proteins of unknown function. Indeed, the protein-protein functional network approach is being used increasingly in the post-genomic era to provide a better understanding of cell functioning and organism development. This functional network is crucial for the systems-level understanding of biological processes and predicting the system’s behavior for the purpose of building a predictive disease model. Previously, we generated functional interaction networks between proteins in MTB strain CDC1551 and used them to predict, where possible, GO biological process terms and functional classes of uncharacterized proteins [2,17], and identified potentially important MTB proteins using network topological properties [29].

Here, we have updated the network to include additional interaction data, predict functions for hypothetical proteins, and analyze the properties of these proteins. Many of these hypothetical proteins have been suspected to play important roles in adhesion of the microbial pathogen in the host system and immune modulation, and also in its intracellular lifestyle, for example, the previously mentioned PE/PPE proteins. The unique sequences in a given organism are often key determinants for species-specific phenotypic properties, such as pathogenicity, and can be interesting drug targets in pathogenic organisms [30]. We believe that this analysis expands our knowledge regarding the functional roles of hypothetical proteins in the MTB system’s behavior.

## 2. Results and Discussion

To investigate the role of MTB hypothetical proteins in the molecular biology of the system, we statistically evaluate the topological values of these proteins compared to the network topological values and to those of other proteins in the MTB protein-protein functional network. Network centrality measures can be used to quantify the role of proteins in the dynamics of the system, e.g., their contribution to the adaptability of the pathogen in the host, enabling the bacterium to colonize host lungs, adapt to their environment, successfully overcome host immune defense, multiply and become numerous enough to cause damage. Furthermore, we evaluated the quality of the annotation predictions and analyzed the biological relevance of the hypothetical proteins using their predicted functional classes and GO biological process terms.

### 2.1. General Topological Parameters of the MTB Functional Network

From the new functional network generated here, a reliability threshold is applied to reduce the impact of bias in functional interactions coming from experimental predictions and computational approaches [29]. Thus, we have only considered those ranging from medium to high confidence, and for functional interactions with low confidence, only those predicted by at least two different approaches were considered. In total, 3 interactions of low confidence predicted by at least two different approaches have been included in the functional network. We determined general properties of this MTB protein functional interaction network (summarized in Table 1).

This new functional network configuration seems to preserve certain topological values compared to the one reported previously, such as the number of hubs, number of connected components and the percentage of proteins in the largest connected component. The functional network exhibits a scale-free topology, *i.e*., the degree distribution of proteins approximates a power law *℘* (*k*) = *k*^−^*^γ^,* with the degree exponent *γ ~* 1.50. Note that here protein hubs are “single points of failure” able to disconnect the functional network. This means that most of the proteins have few interacting partners but some have many partners, allowing any pair-wise protein set in a given connected component to communicate through its relative shortest paths [28,29]. Furthermore, the network has a “small world” architecture, indicating that the transmission of biological information from a given protein to others is achieved through only a few steps (approximately 4), independently of the number of proteins.

Finally, note that even though this novel MTB functional network shares several properties with the previous one in which interologs and functional interactions inferred from protein domain-domain interactions were not included, it fits more perfectly these properties, particularly a “small world” architecture. In this unified configuration the average shortest path length value is 3.6274, which is closer to the order of magnitude log (*n*) [31,32], where log is the decimal logarithm and *n* = 4136 the number of proteins in the network under consideration. This behavior is expected as integrating additional biological evidence into a single network increases the confidence level.

### 2.2. Topological Analysis of MTB Hypothetical Proteins

We used different network centrality scores to check whether the values associated with the hypothetical proteins are significantly higher than network topological values and those of other proteins in the network. The *p*-values in Table 2 were obtained from the *t*-test under the alternative hypothesis that the network topological values of hypothetical proteins are lower than those proteins with known GO biological process terms or the average network topological values, referred to as expected values.

**Figure 1:**
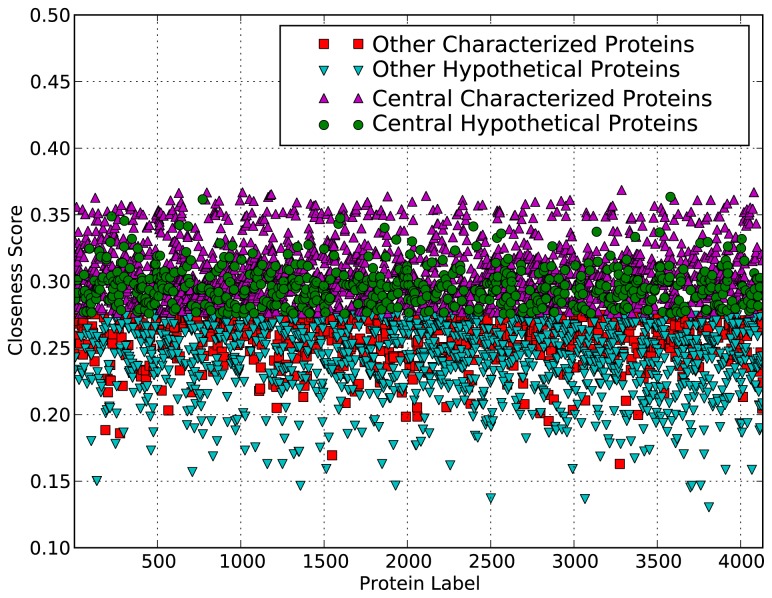
Scatter plot showing proteins that are central in the MTB functional network. Each protein in the genome is plotted by its closeness value and coloured by whether it is characterised and central.

These results indicate that the network topological values of hypothetical proteins tend to be significantly smaller than expected values and network topological values of other proteins. The hypothetical proteins are usually those unique to the organism or environment, so they may not need to be as well connected as other core proteins playing more house-keeping related roles. The low degree may also be due to the limited data available for these proteins. However, for the closeness centrality measure, the closeness score of hypothetical proteins is larger than the expected value. This is also evident from the scatter plot in Figure 1, which shows that many of the hypothetical proteins have high closeness scores compared to the expected value of closeness scores and those of many characterized proteins with GO biological process terms. Closeness score is a measure of the probability that a protein is functionally relevant for several other proteins by bringing proteins or subnetworks together, and does not imply a high degree.

The plot shows which proteins are predicted to be central (above a threshold closeness value). Like the rest of the proteins, the hypothetical proteins’ closeness values are scattered between about 0.17 and 0.36, with 583 of them predicted to be central.

This suggests that some of the hypothetical proteins are in fact potentially important in the MTB functional system and help maintain the “small world” property. Thus, they contribute to ensuring rapid spread of biological information within the system, independently of the system’s complexity. These hypothetical proteins may provide the organism with an evolutionary advantage in the sense that the system would be able to efficiently respond to perturbations in the environment and to quickly exhibit a qualitative change of behaviour in response to these perturbations [29].

We also checked which hypothetical proteins have been shown to be essential for growth [33] or infection [34] in H37Rv. Sixty hypothetical proteins are in this combined essential list, 27 of them were also predicted to be central in the network. This again suggests that some of the hypothetical proteins are important in the lifestyle and survival of the pathogen and thus worthy of further investigation.

### 2.3. Evaluation of Function Predictions

Section 3.2 shows the ROC and P-ROC curves for function prediction algorithm evaluations. As mentioned in this section, we were able to predict functional classes for 82% and GO BP terms for 95% of the uncharacterized proteins. We assessed the validity of predicted GO BP terms by measuring the power of the prediction model to capture GO annotations for these proteins coming from their InterPro matches [35] and corresponding InterPro2GO mappings (both downloaded from the EBI). The correspondence was measured using semantic similarity scores between predicted GO BP terms and those from InterPro matches. The magnitude of this power for a protein *p* is given by

(1) $$s_{p}=\frac{1}{\left|I_{p}\right|}\sum_{t\in T_{p}}S_{GO}\;(t,I_{p})$$

where *T_p_* is the set of predicted GO BP terms of the protein *p*, 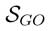 (*t, I_p_*) = max{ 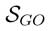 (*t, s*) : *s ∈ I_p_*} with 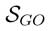 (*t, s*) the GO-universal semantic similarity score between GO terms *t* and *s* [36], and *|I_p_|* stands for the number of GO BP terms in the set $I_{p}=\underset{t\in {\text{IPR}}_{p}}{\cup }{\text{map}}_{GO}(t)$, with IPR*_p_* the InterPro terms of the protein *p* and map*_GO_*(*t*) the set of GO BP terms mapping the InterPro term *t*.

Out of 1770 proteins with predicted GO BP annotations, only 18 proteins have InterPro matches with GO terms. These are likely to be new InterPro matches or mappings, otherwise the GO annotations would already be in the protein GO data. With the prediction annotation model used, InterPro terms of 16 proteins out of 18 have been captured, representing 89%. These proteins together with their matched GO BP annotation predictions, InterPro accessions and their GO mapping, and power scores are shown in Table 3. In this table, the integer beside GO IDs for predictions or InterPro mapping represents their levels in the GO-DAG, with the root of biological process (GO:0008150) assumed to be located at the level 0. In most cases, the power score is 1, indicating that InterPro-associated GO terms of the protein under consideration have also been predicted by the annotation model used. Power scores different from 1.0 are due to different levels of the GO terms identified by InterPro and the prediction. In some cases, InterPro terms are more specific than GO BP annotation predictions and vice versa. It is worth mentioning that GO BP annotation predictions did not match InterPro GO terms for two proteins out of 18, namely P0A5G9 with InterPro entries IPR002145 and IPR010985 corresponding to the same GO BP term “GO:0006355” and O06191 with InterPro entry IPR005471, also mapping to the GO BP “GO:0006355”. The predicted BP terms for both proteins were related to lipid metabolism, while the InterPro mappings are to “regulation of transcription, DNA-dependent” (GO:0006355).

In order to assess the quality of the function predictions, we manually checked the agreement between the function predictions and InterPro matches where available. Of the 1676 proteins that had either functional class or GO BP or both predicted, 937 had InterPro matches, and of these, 621 proteins had matches to entries other than those for PE/PPE, PE-PGRS, Conserved hypothetical protein, Uncharacterised conserved protein, or Domain/Protein of unknown function. The TubercuList functional classes are quite broad, so often the agreement between matches is speculative, but in the majority of cases, functional predictions either agreed with InterPro matches directly or were certainly plausible, based on the protein domains or families matched. In a few cases either the predicted functional class or GO terms agreed with the InterPro matches, but there were very few cases of direct clashes between the predictions and InterPro domains. The InterPro matches for hypothetical proteins identified 6 potential toxin and 6 antitoxin proteins of type II toxin-antitoxin systems, and these were predicted to belong to the virulence, detoxification, adaptation functional class. There are 5 additional antitoxin and 4 toxin proteins in this organism that were already annotated to the virulence, detoxification, adaptation functional class. Other hypothetical proteins predicted to belong to this functional class have GO annotations suggesting involvement in processes such as transcription regulation, lipid metabolism, regulation of growth and response to stress.

### 2.4. Functional Analysis of MTB Hypothetical Proteins and Adaptability

In this section, we look at potential functions that are carried out by hypothetical proteins. Specifically, we are interested in the biological processes in which they are involved, as well as in the functional classes to which they belong in order to assess the potential biological role of these proteins in this pathogen. The distribution of the number of proteins with predicted TubercuList functional classes and statistical significance (*p*-value) of these hypothetical proteins belonging to a given functional class are shown in Table 4. Note that in our context a *p*-value represents the probability of observing the number of predicted proteins in the functional class under consideration by chance, and is computed using formula Equation (4).

Most of them appear to be involved in intermediary metabolism and respiration, as well as in cell wall and cell processes. Almost 72% of these hypothetical proteins are predicted to belong to these two functional classes and a larger number of proteins from the PE/PPE family (100 out of 115 predicted) were assigned to these two classes. The cell wall of MTB plays a key role in its virulence and contributes crucially to the persistence of the pathogen in the host [37–39]. Its unusually low permeability is thought to contribute to the intrinsic drug resistance of mycobacteria [40]. Furthermore, it contributes to the establishment of the interface between the host and pathogen [41], mediating molecular interactions between specific microbial products and host cells. This leads to modification of host cell functioning, thus allowing the parasite to invade host organs and tissues to ensure its survival, and resulting in disease in the host. This, together with the additional 85 virulence-related predictions, suggests that some MTB hypothetical proteins may contribute to the survival and virulence of the pathogen. The fact that a high number of these proteins are predicted to be involved in intermediary metabolism and respiration, suggests that they may provide the pathogen the ability to switch from one metabolic path to another including aerobic and anaerobic, thus allowing the pathogen to survive within the host in different environments ranging from high oxygen potential in the lungs to micro-aerobic/anaerobic conditions within the tuberculous granuloma [17]. These proteins may, therefore, play a role in the persistence of the parasite in the host system.

Finally, we performed GO biological process term enrichment analysis of these hypothetical proteins based on their predicted terms. A summary of the most enriched processes in which these proteins are predicted to be involved are shown in Table 5.

These results show that the majority of newly predicted functions for uncharacterized proteins include basic cellular processes such as transcription, translation, transport, respiration as well as lipid metabolism. Of note is the term “anaerobic respiration” which supports the suggestion above about metabolic flexibility. As mentioned previously, 85 unknown or PE/PPE class proteins were assigned to the virulence, detoxification, adaptation class. In addition, there were 60 proteins already in this class, but with no GO BP terms before function prediction. Most of these 145 proteins were assigned GO terms related to either transcription or lipid metabolism. Two examples include Q7DAC1 and Q7D5E5, putative uncharacterized proteins, both of which are essential, central and predicted to be involved in lipid metabolism, and more specifically, steroid metabolic processes. Q7D5E5, contains a permease domain (IPR003453), which in other proteins is involved in lipid transfer in the cell. These results are in agreement with the suggestion above that these proteins may play an important role in the survival of the MTB pathogen in the host. Many of these hypothetical proteins are unique to MTB or the mycobacteria as they could not be characterized by sequence similarity [17].

## 3. Materials and Methods

We used the MTB protein functional network generated previously [17,29], which includes data from STRING, microarrays, sequence similarity and shared domains, and complemented this with additional interaction data from protein domain-domain interactions and interologs. Protein domain-domain interactions are interactions between proteins whose domains are known or predicted to interact [42]. Interologs are interacting proteins in one organism whose corresponding orthologs also interact in another organism [43]. For predicting interologs, we collected all the interacting pairs of proteins from the IntAct database [44–46] and for each interaction pair, we identified orthologs of both of the proteins constituting the pair in MTB and inferred that these orthologs in MTB also interact. The MTB strain CDC1551 orthologs file was downloaded from the EBI Integr8 project [47,48]. To predict interactions from protein domain-domain interactions, we use known and predicted domain-domain interactions derived from the InterDom database [49,50], which is a database storing putative protein domain-domain interactions. We identified the corresponding domains in MTB and used these to infer new protein-protein interactions. A summary of the number of interactions and confidence scores of the new MTB protein-protein functional interaction network are shown in Table 6.

For scoring protein domain-domain interactions, the predicted interacting domains were considered as domains shared between proteins and we applied the scoring scheme as described in [2]. For interologs, protein-protein interactions from the IntAct database were assumed to be of reasonable quality and we thus set the interologs scores at 0.75. For each evidence source, functional interaction scores are categorized into three different confidence levels, namely low, medium and high confidence. The final row in Table 6 shows the number of interactions in each confidence range for the final combined score computed by combining link confidence score between two proteins *i* and *j* for an integrated view of all datasets through a unified network under assumption of independence, and given by

(2) $$S_{ij}=1-\prod_{d=1}^{3}\left(1-s_{ij}^{d}\right)$$

where *s_ij_^d^* is the confidence score of a functional interaction between *i* and *j* predicted using the type of data *d*. All interactions whose scores are strictly less than 0.3 (*<*0.3) are considered to be low confidence, scores ranging from 0.3 to 0.7 (0.3 *≤ score ≤* 0.7) are classified as medium confidence and scores greater than 0.7 (*>*0.7) yield high confidence. This functional network is used to predict, where possible, functional classes and GO biological process terms of uncharacterized proteins. Furthermore, we compared topological features of these uncharacterized proteins to global network parameters and to those of other proteins, and derived statistically significant annotations among these hypothetical proteins based on the hyper-geometric or binomial distribution model.

### 3.1. Topological Features of the MTB Hypothetical Proteins

To investigate the topological features of hypothetical proteins, we used network centrality measures [29], namely protein degree, closeness, betweenness and eigenvector scores. We compute network centrality scores for all the proteins in the functional network produced using the approach described in [29] as these scores provide an indication of the essentiality of a given protein to the system. The scores for hypothetical proteins are compared to the standard network topological values and to the rest of proteins in the functional network to check whether hypothetical protein scores are significantly higher than network topological values and that of other proteins, *i.e*., whether these hypothetical proteins correspond to bottlenecks in the MTB functional network and thus may be key components of the organism’s cellular processes.

The standard network topological values (see Table 1) are 28.974 for average degree and 3.6274 for average shortest path length. The average closeness score is 1*/*3.6274, which represents approximately 0.27568. For the betweenness centrality measure, the total number of shortest paths expected to pass through the protein in the functional network of interest, is about 15003.

### 3.2. Protein Annotation Prediction

In previous work [17], we suggested the use of the underlying biological principle, referred to as “trace” of the functional network structure under consideration to predict functions of uncharacterized proteins by observing the level 1 and 2 neighbors’ functional annotation occurrence patterns. The approach from [2] was used to predict, where possible, functional classes from TubercuList [51] and GO biological process terms of uncharacterized proteins including PE/PPE proteins.

We denote Level 1, the approach that exploits the guilt-by-association, or level 1 interacting neighbors, to predict the functional class of uncharacterized proteins. The Level 2 approach uses the level 2 interacting neighbors and the Level 1–2 approach combines level 1 and level 2 interacting neighbors to predict the functional class. The Level 1:2 approach uses level 1 neighbors to classify a protein but complemented by level 2 neighbors, used only in the case where level 1 neighbors of the protein under consideration are also uncharacterized, in order to improve coverage.

**Figure 2:**
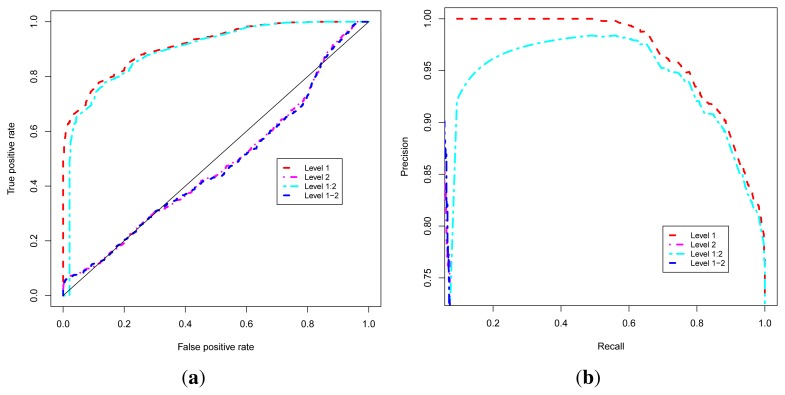
Performance analysis of the functional class prediction approaches. (**a**) ROC curve; (**b**) P-ROC curve.

These four approaches are evaluated using Receiver Operating Characteristic (ROC) [52,53] and Precision-Recall Operating Characteristic (P-ROC) [54] curve analyses and proteins with known functions using the ROCR [55] package under the R programming language [56,57]. In order to compare the performance of these approaches, we combined their related ROC and P-ROC curves, and results are shown in Figure 2a,b. These results indicate that the Level 1 or Guilt-by-association approach yields the best quality prediction, so we used this approach to classify uncharacterized proteins. We were able to predict functional classes for 1466 uncharacterized proteins out of 1784, representing 82% of uncharacterized proteins (unknown + PE/PPE functional classes). This brings the number of proteins with predicted functional classes to 3877 out of 4195 found in the non-redundant list of the MTB proteins from the UniProt database [58–60], which represents 92% of the proteome.

For predicting GO biological process terms, we evaluated five approaches, namely the GO-GA, GO-PIND, GO-GAPIND-1, GO-GAPIND-2 and GO-FS approaches described in [17], with scores and GO semantic similarity computed using GO-universal similarity metrics [36]. The GO-GA approach refers to the Guilt-by-association approach that uses the GO annotation in which relationships between GO terms in the GO directed acyclic graph (GO-DAG) are considered through semantic similarity scores. The GO-GAPIND approaches is a GO annotation prediction model in which the potential annotations of the protein target are annotations occurring among its direct interacting partners and those of other proteins whose direct interacting partners share significant similarity with the set of the direct interacting partners of the protein target. GO-GAPIND-1 uses only level 1 interacting neighbors and GO-GAPIND-2 combines level 1 and 2 interacting neighbors. Finally, the GO-FS approach exploits level-1 and level-2 neighbors similarity weights to identify neighbors that are more likely to share functions with the protein target. Note that all these approaches achieved their best precision at the GO score threshold of 0.1.

**Figure 3:**
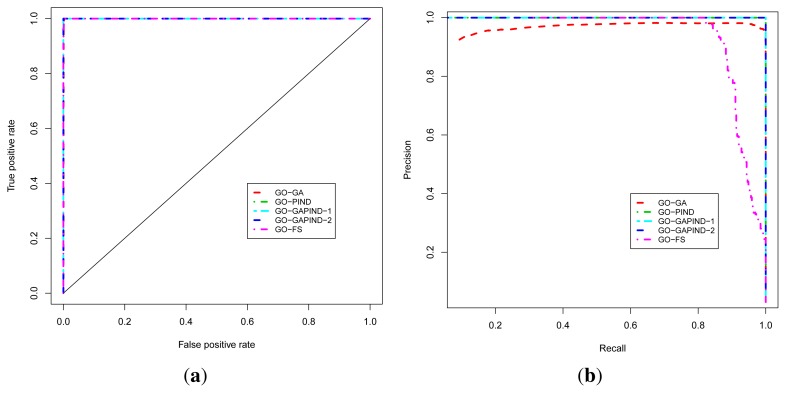
Performance analysis of the function prediction approaches for BP ontology. (**a**) ROC curve; (**b**) P-ROC curve.

The known protein GO annotation data for the MTB proteome were extracted from the Gene Ontology Annotation (GOA) project [11–14] knowing that most of these annotations if not all have been inferred electronically, with IEA as the evidence code for GO. We relied on the fact that the quality of these IEA annotations is high (up to 100% precision and, in the worst case scenario, InterPro2GO, SPKW2GO and EC2GO precisely predict the correct GO term 60 to 70% of the time) [16]. The ROC and P-ROC curves for the five different protein function prediction approaches are depicted in Figure 3a,b, and show that all these approaches achieve good performance in terms of the ROC analysis. To produce these curves, we used leave-one-out cross-validation strategy in which positives for a given known protein are GO terms annotating the protein, and a true positive is any predicted GO term whose semantic similarity score with protein’s known annotations is at least 0.4. Negatives are annotations occurring among a protein’s neighbors whose semantic similarity score with protein’s known annotations is less than 0.4. The P-ROC curves show the difference between these different approaches and reveal that the combination of GO-GA and PIND approaches yields better quality annotations.

In order to ensure higher genome coverage, we ran the prediction model, which uses the GO-GAPIND-2 method to predict GO biological process terms for uncharacterized proteins in the MTB proteome. The GO annotation data extracted from the GOA website contained a total of 2340 proteins characterized with biological process terms. After running the annotation prediction model on the new MTB functional network, the annotations of 1770 proteins out of 1855 uncharacterized proteins were predicted, representing 95% of previously uncharacterized proteins in the MTB proteome. Thus, the resulting annotation dataset consists of 4110 proteins with predicted GO biological process terms, which represents approximately 98% of the whole proteome. Eighty-five proteins are still uncharacterized, representing about 2% of the MTB proteome.

### 3.3. Annotation Enrichment Analysis

Commonly used approaches to assess the statistical significance of term occurrences from a random model include Ficher’s exact test, Chi-squared test, or hyper-geometric and binomial tests. They consist of computing the *p*-value for each term’s frequency in the experiment defined as the probability that the number of genes annotated with the term under consideration in the target set occurs by chance or is comprised of randomly drawn genes. These different tests are performed under the null hypothesis that the number of genes annotated by the term under consideration have the same probability of falling in the reference set and in the target set. Fisher’s exact and Chi-squared tests are non-parametric and less powerful than binomial and hyper-geometric tests [61].

Using the hyper-geometric distribution, the *p*-value, which is the probability of observing at least ℓ genes from a target gene set of size *n* by chance, knowing that the reference dataset, considered to be a background distribution, contains *m* such annotated genes out of N genes is given by

(3) $$P\;[X\geq \ell ]=1-\sum_{k=0}^{\ell -1}\frac{\left(\begin{array}{c} m\\ k \end{array}\right)\left(\begin{array}{c} N-m\\ n-k \end{array}\right)}{\left(\begin{array}{c} N\\ n \end{array}\right)}$$

where the random variable *X* represents the number of genes within a given gene subset, annotated with a given term. For the large sample size, the hyper-geometric distribution converges to a binomial distribution [62], in which case the *p*-value is computed as follows:

(4) $$P\;[X\geq \ell ]=1-\sum_{k=0}^{\ell -1}\left(\begin{array}{c} n\\ k \end{array}\right)p^{k}{\left(1-p\right)}^{n-k}$$

where the probability *p* is estimated by the relative frequency of occurrence of each GO term in the reference dataset. In these two cases, the lower the *p*-value, the less likely it is that the observed frequency of the term is due to chance, and thus the more meaningful the term is in the target gene set. The implementation of *p*-value allows an automatic ranking of all terms in the dataset under consideration. This was used for enrichment analysis on the newly predicted functions for the hypothetical proteins.

## 4. Conclusions

The existence of hypothetical proteins in genomes constitutes a major issue for comparative and functional genomics analyses. In particular for pathogenic organisms, these hypothetical proteins hamper the search for new and effective drug targets, and weaken progress towards the advancement of research on these organisms and enhancement of our understanding of their virulence and pathogenicity. In this study, we used network topological scores and predicted annotations of the MTB hypothetical proteins from a protein-protein functional network to elucidate potential roles of these proteins in the functioning of the system. This was achieved by performing statistical analysis of network topological scores of these proteins and annotation enrichment analysis of predicted annotations for these proteins.

We showed that in the context of MTB, some of these proteins may contribute to the survival of the bacterial pathogen within the host system and they may have a particular role in helping the organism evade the host immune response and in persistence and latency. They are thus likely to be important for the specific lifestyle of the organism and adaptability of this pathogen in the host, so functional characterization of these proteins is essential. Currently, there is a need for novel and effective drugs with new biological mechanisms of action against drug susceptible and drug-resistant strains. These need to be reliably administered with a shorter regimen to overcome the disease caused by this particular organism, which constitutes a public health challenge, claiming millions of lives and new cases every year. Such quantitative analysis may help us better understand the biology of the organism as a whole system and identify potential drug targets at the molecular level for the disease.

## Figures and Tables

**Table 1:** General MTB functional network topological parameters.

Parameters	Value
Number of Proteins (Nodes)	4136
Number of Functional Interactions (Edges)	59,919
Average Degree (in and out)	28.974
Average Shortest Path Length	3.6274
Maximum Path Length	11
Number of Connected Components	23
% of Nodes in Largest Component	98.7%
Number of Hubs	201

**Table 2:** Comparison of network topological properties of hypothetical proteins to the standard network topological values and those of proteins with previously known GO biological process terms.

Metric	Average values			*p*-values	

	Hypothetical	Other proteins	Expected value	Other proteins	Expected value
Degree	16.16892	37.77267	28.09381	*<* 2.2 *×* 10^−16^	*<* 2.2 *×* 10^−16^
Closeness	0.277673	0.293277	0.27568	5.562 *×* 10^−08^	0.9792
Betweenness	6948.217	13913	15003	*<* 2.2 *×* 10^−16^	*<* 2.2 *×* 10^−16^
Eigenvector	0.000314	0.00591	0.00340	*<* 2.2 *×* 10^−16^	*<* 2.2 *×* 10^−16^

**Table 3:** Proteins that have InterPro matches mapped to GO terms and their similarities to predicted GO terms.

UniProt-ID	InterPro-ID	GO mapping	GO Prediction	Power
P67745	IPR000835	GO:0006355 [8]	GO:0006355 [8]	1.00000
Q8VKE6	IPR002514	GO:0006313 [8]	GO:0006313 [8]	1.00000
Q7D8E8	IPR001845	GO:0006355 [8]	GO:0006355 [8]	1.00000
Q7D5V1	IPR000836	GO:0009116 [6]	GO:0009116 [6]	1.00000
Q7D8W2	IPR001087	GO:0006629 [3]	GO:0006629 [3]	1.00000
Q8VJC1	IPR006059	GO:0006810 [3]	GO:0006810 [3]	1.00000
Q7D9M5	IPR020946	GO:0055114 [2]	GO:0055114 [2]	1.00000
P64725	IPR002539	GO:0008152 [1]	GO:0008152 [1]	1.00000
P71788	IPR013216	GO:0008152 [1]	GO:0008152 [1]	1.00000
Q10777	IPR000873	GO:0008152 [1]	GO:0008152 [1]	1.00000
O05796	IPR013216	GO:0008152 [1]	GO:0008152 [1]	1.00000
O07197	IPR013094	GO:0008152 [1]	GO:0008152 [1]	1.00000
P0A5F5	IPR005674	GO:0008152 [1]	GO:0008152 [1]	0.66604
	IPR000383	GO:0006508 [4]	GO:0044238 [2]	
O06547	IPR013216	GO:0008152 [1]	GO:0044237 [2]	0.24819
Q7D8C2	IPR013216	GO:0008152 [1]	GO:0042158 [5]	0.03033
O05294	IPR012908	GO:0006886 [6]	GO:0044237 [2]	0.01435
		GO:0006505 [8]		

**Table 4:** Repartition per class of hypothetical proteins in the MTB proteome before and after function prediction. PE/PPE- indicates the number of predicted proteins originating from the PE/PPE family.

Functional Class	# Proteins before	Prediction	PE/PPE-	*p*-values	# Proteins after	% change
1 virulence, detoxification, adaptation	176	85	1	3.33067 *×* 10^−16^	261	32.6
2 lipid metabolism	230	80	6	9.76996 *×* 10^−15^	310	25.8
3 information pathways	245	93	3	9.76996 *×* 10^−15^	338	27.5
4 cell wall and cell processes	618	418	62	3.33067 *×* 10^−16^	1036	40.3
5 insertion seqs and phages	82	65	4	1.11022 *×* 10^−16^	147	44.2
6 PE/PPE	147	−115	-	-	32	-
7 intermediary metabolism and respiration	884	637	38	4.95160 *×* 10^−14^	1521	41.9
8 unknown	1637	−1351	-	-	286	
9 regulatory proteins	176	88	1	9.54792 *×* 10^−15^	264	33.3

Total	4195	1466	115	-	4195	37.8

**Table 5:** GO process terms significantly over-represented in the newly predicted GO set compared to complete set of GO terms.

GO ID	GO name	Frequency	*p*-Value
GO:0006730	one-carbon metabolic process	364	0.00000
GO:0009132	nucleoside diphosphate metabolic process	74	0.00000
GO:0009123	nucleoside monophosphate metabolic process	72	0.00000
GO:0009141	nucleoside triphosphate metabolic process	71	0.00000
GO:0006353	transcription termination, DNA-dependent	88	1.11022 *×* 10^−16^
GO:0019538	protein metabolic process	87	1.11022 *×* 10^−16^
GO:0022900	electron transport chain	354	2.22045 *×* 10^−16^
GO:0006793	phosphorus metabolic process	324	2.22045 *×* 10^−16^
GO:0009061	anaerobic respiration	135	2.22045 *×* 10^−16^
GO:0009307	DNA restriction-modification system	73	2.22045 *×* 10^−16^
GO:0006662	glycerol ether metabolic process	277	3.33067 *×* 10^−16^
GO:0015074	DNA integration	157	3.33067 *×* 10^−16^
GO:0019419	sulfate reduction	86	3.33067 *×* 10^−16^
GO:0051090	regulation of sequence-specific DNA binding transcription factor activity	64	3.33067 *×* 10^−16^
GO:0006139	nucleobase-containing compound metabolic process	336	4.44089 *×* 10^−16^
GO:0006259	DNA metabolic process	169	4.44089 *×* 10^−16^
GO:0006313	transposition, DNA-mediated	113	4.44089 *×* 10^−16^
GO:0006797	polyphosphate metabolic process	672	5.55112 *×* 10^−16^
GO:0006796	phosphate-containing compound metabolic process	643	5.55112 *×* 10^−16^
GO:0000103	sulfate assimilation	603	5.55112 *×* 10^−16^
GO:0044238	primary metabolic process	548	5.55112 *×* 10^−16^
GO:0006281	DNA repair	246	5.55112 *×* 10^−16^
GO:0006413	translational initiation	156	5.55112 *×* 10^−16^
GO:0009116	nucleoside metabolic process	107	5.55112 *×* 10^−16^
GO:0044255	cellular lipid metabolic process	444	6.66134 *×* 10^−16^
GO:0044262	cellular carbohydrate metabolic process	317	6.66134 *×* 10^−16^
GO:0001121	transcription from bacterial-type RNA polymerase promoter	273	6.66134 *×* 10^−16^
GO:0006302	double-strand break repair	200	6.66134 *×* 10^−16^
GO:0009225	nucleotide-sugar metabolic process	177	6.66134 *×* 10^−16^
GO:0006310	DNA recombination	170	6.66134 *×* 10^−16^
GO:0006260	DNA replication	156	6.66134 *×* 10^−16^
GO:0006396	RNA processing	109	6.66134 *×* 10^−16^
GO:0015977	carbon fixation	534	7.77161 *×* 10^−16^
GO:0042126	nitrate metabolic process	477	7.77156 *×* 10^−16^
GO:0006082	organic acid metabolic process	244	7.77156 *×* 10^−16^
GO:0006266	DNA ligation	150	7.77156 *×* 10^−16^
GO:0006104	succinyl-CoA metabolic process	144	7.77156 *×* 10^−16^
GO:0009060	aerobic respiration	118	7.77156 *×* 10^−16^
GO:0006352	transcription initiation, DNA-dependent	112	7.77156 *×* 10^−16^
GO:0044249	cellular biosynthetic process	618	8.88178 *×* 10^−16^
GO:0006351	transcription, DNA-dependent	333	8.88178 *×* 10^−16^
GO:0009399	nitrogen fixation	271	8.88178 *×* 10^−16^
GO:0016042	lipid catabolic process	107	8.88178 *×* 10^−16^
GO:0009117	nucleotide metabolic process	101	8.88178 *×* 10^−16^
GO:0043620	regulation of DNA-dependent transcription in response to stress	96	8.88178 *×* 10^−16^
GO:0001522	pseudouridine synthesis	92	8.88178 *×* 10^−16^
GO:0006314	intron homing	137	9.99201 *×* 10^−16^
GO:0006066	alcohol metabolic process	635	1.11022 *×* 10^−15^
GO:0090305	nucleic acid phosphodiester bond hydrolysis	222	1.11022 *×* 10^−15^
GO:0006284	base-excision repair	212	1.11022 *×* 10^−15^
GO:0006289	nucleotide-excision repair	210	1.33227 *×* 10^−15^
GO:0009451	RNA modification	101	1.33227 *×* 10^−15^
GO:0008610	lipid biosynthetic process	184	1.66534 *×* 10^−15^
GO:0044267	cellular protein metabolic process	58	3.66374 *×* 10^−15^
GO:0006268	DNA unwinding involved in replication	53	7.32747 *×* 10^−15^
GO:0006270	DNA-dependent DNA replication initiation	52	1.36557 *×* 10^−14^
GO:0006412	translation	207	4.56302 *×* 10^−14^
GO:0031554	regulation of transcription termination, DNA-dependent	50	5.19584 *×* 10^−14^
GO:0030261	chromosome condensation	56	1.21125 *×* 10^−13^
GO:0006801	superoxide metabolic process	693	2.91767 *×* 10^−13^
GO:0000725	recombinational repair	46	8.03135 *×* 10^−13^

**Table 6:** The number of associations in the MTB functional network, shown separately for each data source and confidence range from low to high.

Association Evidence by Type	Low Confidence	Medium Confidence	High Confidence
Previous Functional Network	6850	32488	25605
Domain-domain	0	5082	864
Interologs	0	0	1701
Combined Score	6844	30142	29776
